# En bloc giant cell tumor resection following direct hemiarthroplasty shoulder reconstruction–functional outcome: A case report

**DOI:** 10.1016/j.ijscr.2019.07.052

**Published:** 2019-07-26

**Authors:** Muhammad Andry Usman, Andi Dhedie Prasatia Sam, Marcell Wijaya, Roichan Muhammad Firdaus, Khrisna Yudha

**Affiliations:** a Orthopaedic and Traumatology Department, Hasanuddin University, Makassar, Indonesia; b Consultant of Orthopaedic and Traumatology Department, Hasanuddin University, Makassar, Indonesia

**Keywords:** Giant cell tumor, Proximal humerus, En bloc resection, Hemiarthoplasty, Cementless endoprosthetic implant

## Abstract

**Introduction:**

Giant cell tumor is a type of benign tumor which has the characteristic of rapidly growing and a chance to metastasis. It is however locally aggressive and would typically affect young patients. They commonly present with pain and associated with pathological fracture.

**Presentation of case:**

This is an uncommon case of 29 years old male with pathological fracture and giant cell tumor in proximal humerus. A plain radiograph revealed pathological fracture in head of humerus and histopathology examination was consistent with giant cell tumor. The patient had surgical option with en bloc giant cell tumor resection following hemiarthroplasty with cementless endoprosthetic implant for humerus, which aimed to provide a single step surgery without any interval debulking surgery. The patient had achieved bony union between 6 weeks after the surgery and recurrence was not found by the time of the last follow-up.

**Discussion:**

Based on Campanacci’s classification the tumor is divided into 3 stages. The management of giant cell tumors continues to be one of the most challenging areas in orthopedic oncology. Surgery is the first line option; however, it is depending on the tumor staging and can vary from intralesional curettage to total resection of the tumor. Since the local behavior of giant cell tumors has a high risk of local recurrence, en bloc resection and reconstruction were chosen for these Grade III lesions.

**Conclusion:**

The aim of this procedure is to preserve the shoulder joint shown satisfaction in a clinical, radiological, functional and esthetic result.

## Highlights

• Giant cell tumor is locally aggressive and destructive to the affected area.
• Reconstruction after tumour resection is challenging.
• Single stage surgery is performed and give satisfactory result.

## 1 Introduction

Giant cell tumor (GCT) is a type of benign tumor which is locally aggressive and has the capability to metastasize [[1], [2], [3], [4], [5], [6], [7], [8], [9], [10], [11], [12], [13], [14]]. It represents approximately 5% of all primary bone tumors [2,[2], [3], [4], [5], [6], [7], [8], [9], [10], [11], [12], [13], [14]]. 85% lesion occur in long bones with most frequent sites in distal femur (26%), proximal tibia (19%) and distal radius (11%) [[1], [2], [3], [4], [5], [6], [7], [8],^10^,[2], [3], [4], [5], [6], [7], [8], [9], [10], [11], [12], [13], [14]]. Giant cell tumor in the proximal humerus is accounted for 4% of all giant cell tumor cases [8]. Pain and functional disability are the major reason for hospital visit [4,6,8,9,14].

The tumor is categorized based on radiograph appearance using the classification by Campanacci and is divided into 3 stages. Grade 1 tumor has well-marginated border of thin rim of mature bone with intact and non-deformed cortex. Grade 2 tumor still has well-defined margins but no radiopaque rim. Cortex is thin and moderately expanded. Grade 3 tumor has ill-defined border and permeates into surrounding soft tissue, with discontinuity of cortical bone [[5], [6], [7],^14^].

Treatment of choice is surgery, either intralesional curettage or wide resection, depending on the staging of tumor [[2], [3], [4], [5], [6], [7], [8], [9], [10], [11], [12], [13], [14]]. Recurrence rate can be up to 50% with curettage and as low as 20% with wide resection [[2], [3], [4], [5], [6], [7], [8], [9], [10], [11], [12], [13], [14]].

This work has been reported in line with the SCARE criteria [15].

## 2 Presentation of case

**Fig. 1:**
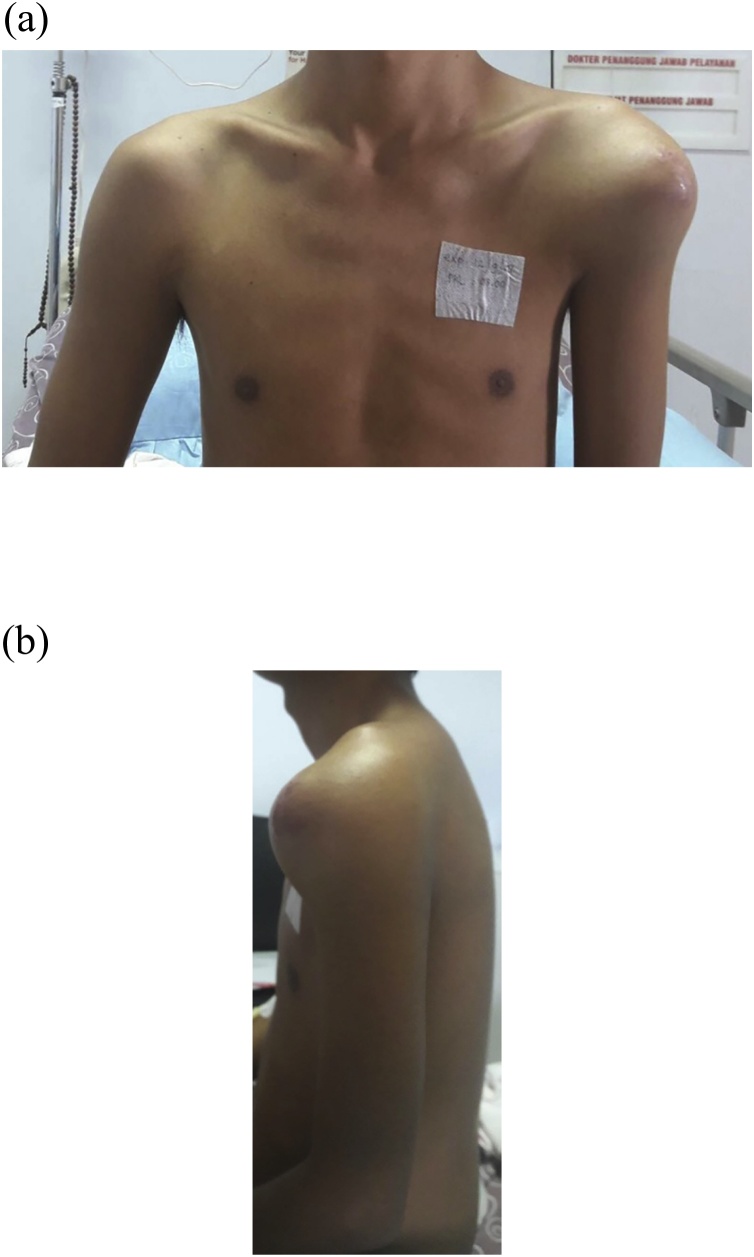
(a) Front aspect of shoulder mass with significant difference compared to contralateral aspect. (b) Lateral aspect of shoulder mass. Frontal enlargement and a visivle erythema can be seen on the frontal aspect of the shoulder.

**Fig. 2:**
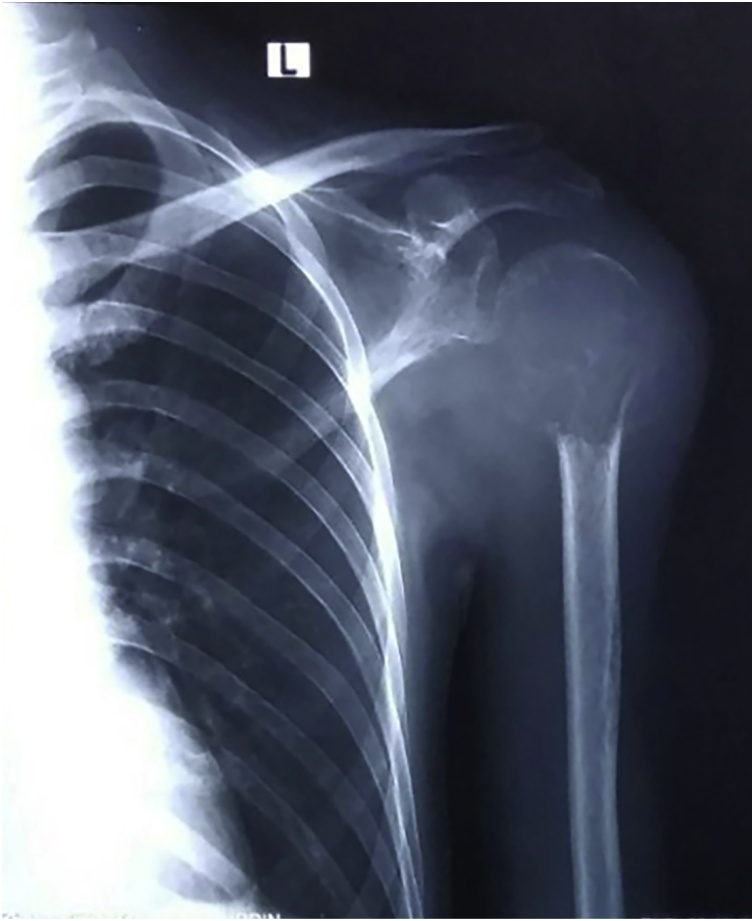
Radiograph of the left shoulder. An expansile and osteolytic lesion was visible with thinning of cortex in the proximal humerus.

**Fig. 3:**
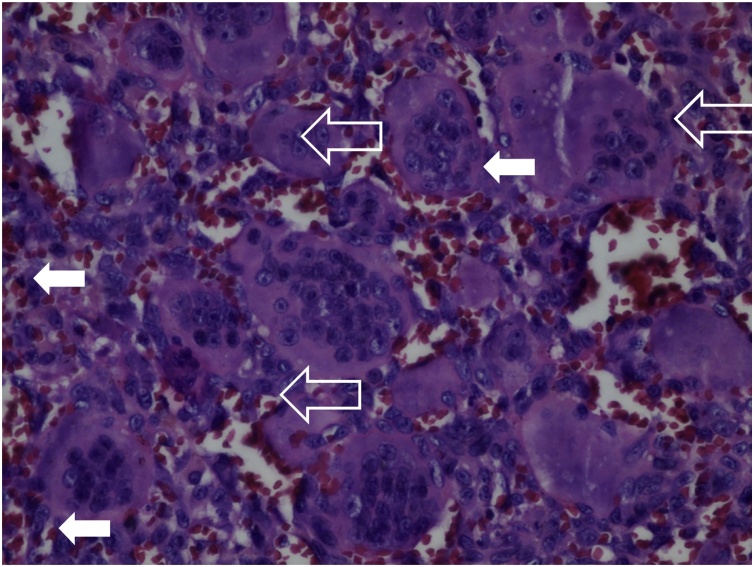
Histopathologic section of Giant Cell tumor.

A 29-year old male was reffered from other general hospital to our outpatient clinic with left shoulder rapid growing mass and pain during activities in 3 months. The patient had loss of shoulder function with range of motion (ROM) was 20° of flexion and abduction was limited to 15°, internal or external rotation was unable to be performed due to pain. On physical examination, a mass was visible in the left shoulder (Fig. 1). The mass was fixed with smooth wall defined border. Local pain was present, and skin temperature was higher. Neither bruit nor venectation was present. Palpation of axillary area revealed no lymph node enlargement. Plain shoulder radiographs showed expansive and osteolytic lesion with thin cortex in the proximal humerus and discontinuity in some areas (Fig. 2). Routine blood examination showed normal range of Erythrocyte sedimentation rate, C-reactive protein, Lactate dehydrogenase, alkaline phosphatase, and total serum calcium. Open biopsy was performed and histopathological features indicated large giant cells containing multiple nuclei mixed with round mononuclear cells and neoplastic spindle mononuclear cells can be seen in Fig. 3. the patient had no prior medical illness. Different reconstruction options were considered included proximal humerus reconstruction with vascularized proximal fibular autograft and two-stages surgery with megaprosthesis reconstruction. However, taking into consideration of the time efficacy and surgical effectiveness, a standard total shoulder replacement with hemiarthroplasty was planned with a cementless porous coated humeral stem implant. However, we had proximal fibular autograft as backup option. The precise margins of the tumor were ascertained using plain radiography. A wide margin excision of 2 cm from the reactive zone were performed [16].

**Fig. 4:**
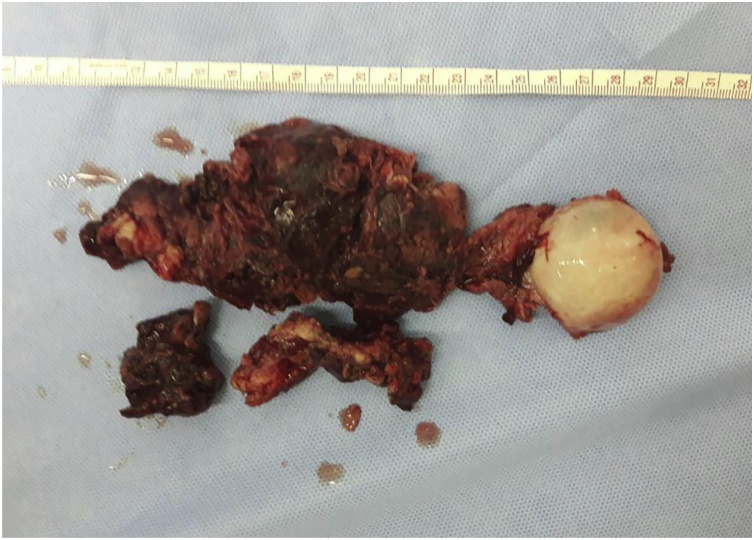
Macro section of tumor after en-bloc resection showing the tumor, head of humerus and a portion of the normal bone.

**Fig. 5:**
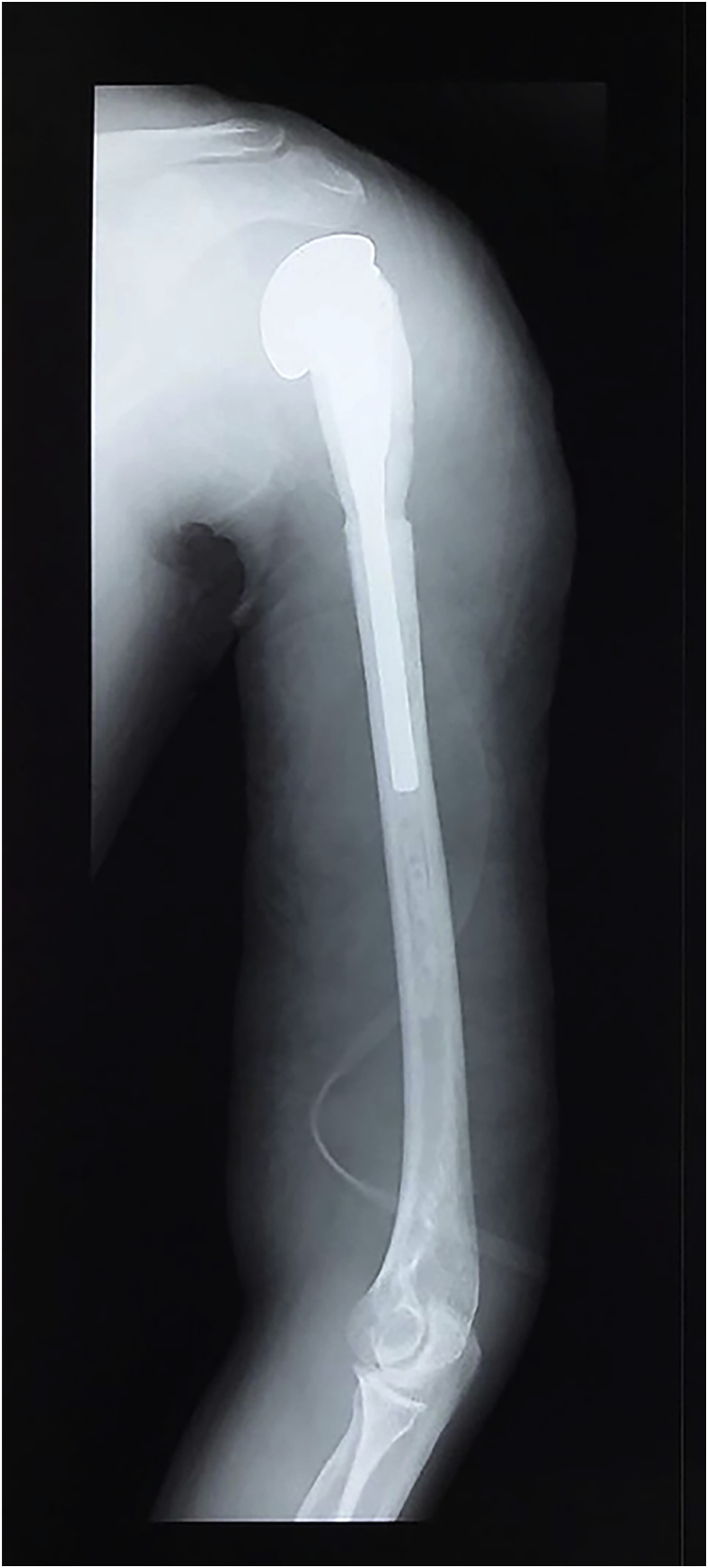
Post-operative radiograph of the left shoulder.

The resection site of GCT in the proximal humerus was approached directly via a delto-pectoral incision. During the rotator cuff muscle detachment, the meticulous hemorrhage control of anterior circumflex humeral artery, posterior circumflex humeral artery and cephalic vein to reach tumor site and tumor en-bloc resection was performed (Fig. 4). After the free-tumor area was confirmed by way of macroscopic and pathologic confirmation, the cementless endoprosthetic implant stem was reamed into the intramedullary canal of distal part of humerus. The head of the implant was placed in the glenohumeral articulation joint (Fig. 5). The rotator cuff muscles were reattached above the implant body with polyester suture. The ROM was performed to assess the stability of glenohumeral joint. Afterward, alcohol was applied meticulously with a surgical cotton ball at a concentration of 90%, after which the cavity was irrigated. This cycle was repeated three times, followed by pulse irrigation with distilled water. The surgical site was closed with 3-0 monofilament interrupted suture. Antibiotic administered for 3 days and bisphosphonate for bone growth was given 3 months postoperatively.

**Fig. 6:**
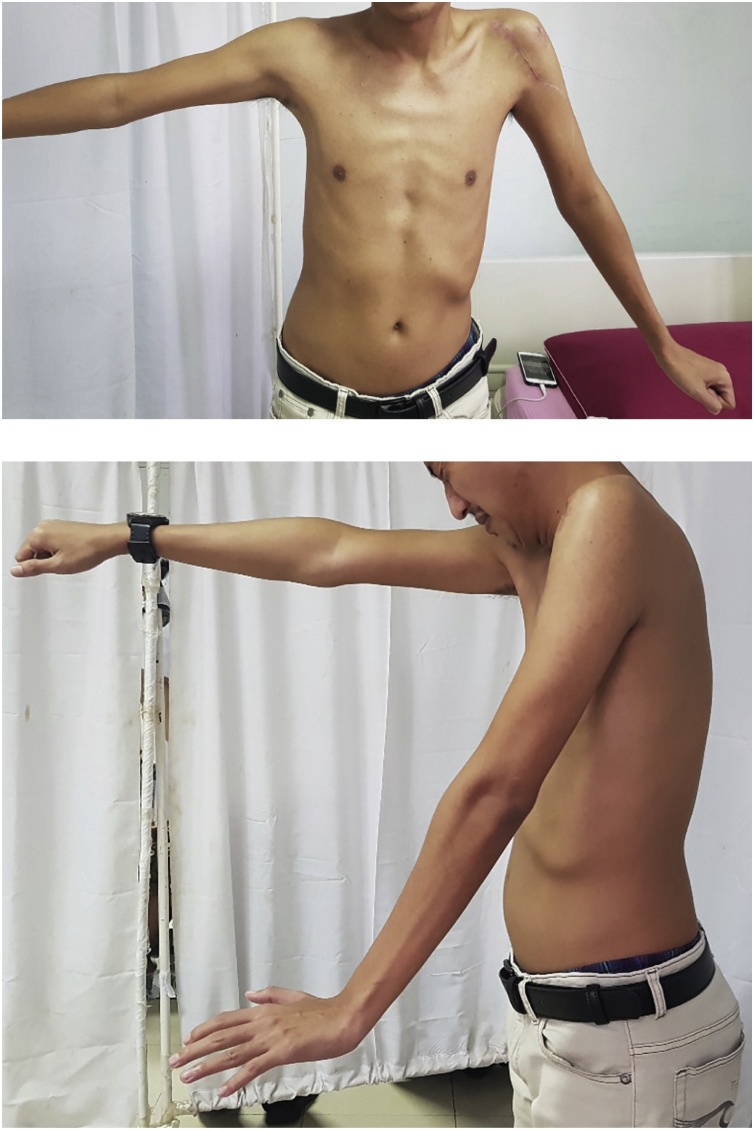
Range of Motion and clinical outcome at 3 months post operative.

**Fig. 7:**
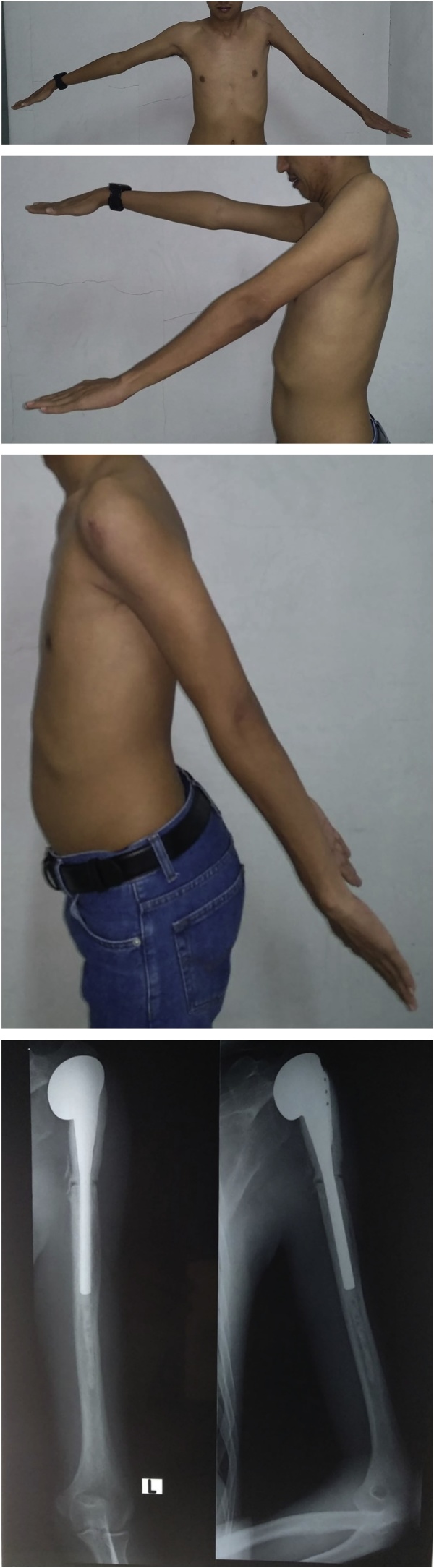
Clinical Outcome and post operative radiograph at 6 months post operative.

Follow-ups were done on 3^rd^ (Fig. 6) and 6^th^ (Fig. 7) months to evaluate the function of left shoulder. Patient had achieved bony union between 6 weeks after the surgery. Signs of GCT recurrence was not yet found by the time of the last follow-up. Neither Clinical assessments nor conventional chest radiograph has revealed any presence of metastases. Chest computed tomography scan, although sensitive in detecting pulmonary metastases, were unable to be performed due to limitations of medical equipment. Patient had no discomfort at the surgical site nor complained of instability of the shoulder. Neither postoperative infections and neurovascular complications occurred in the patient.

Rehabilitation was started at 3^rd^-week postoperative. The flexion-extension range was improved between 45° and 30°. The physiotherapeutic evaluation consisted of assessing the pain, analyzing the motion and making an anthropometric evaluation. Radiographic evaluation on the operated shoulder showed preservation of the joint relations and incorporation of implant to the humeral end. The total musculoskeletal tumor society (MSTS) score after surgery is 19.

## 3 Discussion

Giant cell tumors (GCT) are benign, locally aggressive tumors, typically affecting young patients. They commonly present with pain and 10–15% have an associated pathological fracture [11]. The management of giant cell tumors continues to be one of the most challenging areas in orthopedic oncology. Since the local behavior of giant cell tumors can be aggressive and they have a high risk of local recurrence of up to 25%, en bloc resection and reconstruction were chosen for these Grade III lesions from the point of view of preventing local recurrence rate and preserving joint [17,18]. High tendency of GCT reccurence occurs within the first 24 months, and recurrency of the tumor were absent during the 6 months follow up period [4]. It’s indeed too early to detect the signs of tumor recurrence in just 6 month-period since the local recurrence may appear within 24 months.

Campanacci’s radiographic classification for surgical staging was helpful to initiate the surgical option. However, there are several surgical procedures that can be done including proximal fibular autograft, megaprosthesis reconstruction, and endoprosthetic replacement. Proximal fibular autograft has disadvantages, including lack of blood supply and osteogenic cells, potential immunologic reactions, and possibility of collapse secondary to bone allograft absorption. Therefore, bone allografts are not the best choice for reconstruction and do not result in very satisfactory outcomes [13]. The use of megaprosthetic implant gained the worldwide recognition for tumor reconstruction, however, due to the large portion of implant and major resection of the tumor, the surgery needs free flap for soft tissue coverage [19].

Endoprosthetic replacement is used in salvaging the proximal humerus, and some reports indicate that the replacement is associated with a low complication rate and high implant survival rate [20,21]. The patient with a giant cell tumor of the present report had a rapid growing of the tumor and non-intact periosteum. It was decided to preserve the patient’s joint surface by means of segmental resection and the use of an endoprosthetic for the humerus because of his youth, the longevity observed among patients with this type of tumor and the possibility of successive surgical interventions to which the patient would be subjected if an endoprosthetic replacing the shoulder joint surface were to be implanted. Our post-operative result suggests that patients with intraarticular resection with endoprosthetic replacement of the proximal humerus have a preserved axillary nerve, rotator cuff, good active shoulder ROM, and stable joint. The MSTS score for shoulder including the motion, pain, stability, deformity, strength, functional activity, and emotional acceptance also showed improvement.

The 6 months duration of the follow up represented the final result of functional outcome as the implant had started to be incorporated in the arm and soft tissue had already entered remodelling phase.

## 4 Conclusion

A case which were consistent with Campanacci 3 Giant Cell tumor presented in this study which were treated with en bloc resection and hemiarthroplasty. The use of cementless endoprosthetic implant for humerus to preserved the shoulder joint shown satisfaction in clinical, radiological, functional and esthetic result. Further prospective studies including complete series of examination consist of more advanced radiographic study with long-term follow-up should be conducted.

## Sources of funding

No sources of funding is received from any sponsors.

## Ethical approval

This study was approved by the ethical board of Hasanuddin University of Makassar.

Ethic approval number 368 / H4.8.4.5.31 / pp36-kometik / 2018.

## Consent

Written informed consent was obtained from the patient for publication of this case report and accompanying images. A copy of the written consent is available for review by the Editor-in-Chief of this journal on request.

## Author’s contribution

Muhammad Andry Usman was the major contributor of idea of the manuscript (concept design, data analysis and interpretation) and act as the first writer.

Andi Dhedie Prasatia was the operator of the operation and act as the second writer.

Marcell Wijaya contributed to data collection, analysis and interpretation of data, manuscript drafting and revision, and acted as the corresponding author.

Roychan Firdaus contributed to rehabilitation care of the patient.

Khrisna Yudha help contributed to data collection and revision of the manuscript.

All author reviewed and approved the final manuscsript.

## Registration of research studies

Not applicable.

## Guarantor

Marcell Wijaya.

## Provenance and peer review

Not commissioned, externally peer-reviewed.

## Declaration of Competing Interest

No conflict of interest.
